# Filtering Entities to Optimize Identification of Adverse Drug Reaction From Social Media: How Can the Number of Words Between Entities in the Messages Help?

**DOI:** 10.2196/publichealth.6577

**Published:** 2017-06-22

**Authors:** Redhouane Abdellaoui, Stéphane Schück, Nathalie Texier, Anita Burgun

**Affiliations:** ^1^ INSERM UMRS 1138 Team 22 Université Pierre et Marie Curie Paris France; ^2^ Kappa Santé Innovation Paris France; ^3^ Assistance Publique-Hôpitaux de Paris (AP-HP), Hôpital Européen Georges-Pompidou (HEGP) Medical Informatics Paris France

**Keywords:** pharmacovigilance, social media, text mining, Gaussian mixture model, EM algorithm, clustering, density estimation

## Abstract

**Background:**

With the increasing popularity of Web 2.0 applications, social media has made it possible for individuals to post messages on adverse drug reactions. In such online conversations, patients discuss their symptoms, medical history, and diseases. These disorders may correspond to adverse drug reactions (ADRs) or any other medical condition. Therefore, methods must be developed to distinguish between false positives and true ADR declarations.

**Objective:**

The aim of this study was to investigate a method for filtering out disorder terms that did not correspond to adverse events by using the distance (as number of words) between the drug term and the disorder or symptom term in the post. We hypothesized that the shorter the distance between the disorder name and the drug, the higher the probability to be an ADR.

**Methods:**

We analyzed a corpus of 648 messages corresponding to a total of 1654 (drug and disorder) pairs from 5 French forums using Gaussian mixture models and an expectation-maximization (EM) algorithm **.**

**Results:**

The distribution of the distances between the drug term and the disorder term enabled the filtering of 50.03% (733/1465) of the disorders that were not ADRs. Our filtering strategy achieved a precision of 95.8% and a recall of 50.0%.

**Conclusions:**

This study suggests that such distance between terms can be used for identifying false positives, thereby improving ADR detection in social media.

## Introduction

### Background

Adverse drug reactions (ADRs) cause millions of injuries worldwide each year and require billions of Euros in associated costs [1,2]. Drug safety surveillance targets the detection, assessment, and prevention of ADRs in the postapproval period. A promise of augmenting drug safety with patient-generated data drawn from the Internet was called for by several scientific committees related to pharmacovigilance in the United States and in Europe [3,4].

There are now sites for consumers that enable patients to report ADRs. Patients who experience ADRs want to contribute drug safety content, share their experience, and obtain information and support from other Internet users [5-8].

Three recently published review articles showed that the use of social media data for ADR monitoring was increasing. Sarker et al analyzed 22 studies that used social media data. They observed that publicly available annotated data remained scarce, thus making system performance comparisons difficult [9]. Golder et al analyzed 51 studies based on a total of 174 social media sites, most of which had discussion forums (71%). They used broad selection criteria and considered several types of social media including messages, social networks, patient forums, Twitter, blogs, and Facebook [10]. Ninety percent (45/51) of the papers looked for any adverse events, and 10% (5/51) focused on specific adverse events (eg, fatal skin reactions or hypersensitivity). The overall prevalence of adverse event reports in social media varied from 0.2% to 8% of the posts. There was general agreement that a high frequency of mild adverse events was identified but that the more serious events and laboratory-based ADRs were under-represented in social media. Lardon et al explored methods for identifying and extracting target data and evaluating the quality of medical information from social media. Most studies used supervised classification techniques to detect posts containing ADR mentions and lexicon-based approaches to extract ADR mentions from texts [9,11].

When the methods relied on the development of lexicons, these studies were generally limited in the number of drugs studied or the number of target ADRs. For example, Benton et al focused on 4 drugs [12]; Yang et al focused on 10 drugs and 5 ADRs [13]; Yates et al focused on breast cancer-associated ADRs [14]; Jiang et al focused on 5 drugs [15]; and Sarker and Gonzalez focused on various drugs prescribed in chronic diseases, such as type 2 diabetes [16].

Other authors focused on detecting user posts mentioning potential ADRs. Some of them combined social media with other knowledge sources such as Medline [17]. The binary classification of text into ADR versus non-ADR categories has been typically performed in previous research work using three supervised classification approaches: (1) Naïve Bayes (NB), (2) support vector machine (SVM), and (3) maximum entropy (ME). Among those, SVMs are the most popular for text classification tasks [18], including ADR text.

In 13 studies using automatic processing based on data mining to analyze patient declarations, 7 studies aimed at identifying the relationships between disease entities and drug names. Five of these studies used machine learning methods. Qualitative analyses of forums and mailing list posts show that it may be used to identify rare and serious ADRs (eg, [11,19,20]) and the unexpected frequency of known ADRs. However, the use of social media for data source pharmacovigilance must be validated [10].

Therefore, the main challenge lay in identifying a combination of methods that could reduce the overall number of misclassifications of potential ADRs from patient’s posts. In all such studies, the authors analyzed messages that contained references to both a drug and a disorder or symptom. ADRMine, a machine learning–based concept extraction system [21] that uses conditional random fields (CRFs), achieved an *F* measure of 0.82 in the ADR extraction task.

However, ADR messages from social media are not only factual descriptions about adverse events [10]. The messages may also include contextual information (the patient’s condition and comorbidities) and opinions and feelings about treatments and drugs (eg, providing personal experience about a treatment, discussing new research, explaining documentation and drug monograph to a peer, and exchanging information relevant to patient’s daily lives).

Before robust conclusions can be drawn from social media regarding ADRs, the biggest problem with automated or semiautomated methods is distinguishing between genuine ADRs and other types of cooccurrence (eg, treatments and context) between drugs and diseases in messages. To quote Golder [10], “the purported adverse events may not be adverse events at all. Terms used to describe adverse events can also be used for indications of the condition being treated (eg, confounding by indication), beneficial effects (ie, sleepiness can be a beneficial effect for someone with insomnia), or may not have been experienced by a patient.” This notion can be illustrated by an article published by Benton et al [12]. The authors analyzed social media to identify adverse events that were associated with the most commonly used drugs to treat breast cancer. In their study, “uterine cancer” cooccurred 374 times with tamoxifen. However, most of the messages involved anxiety about taking tamoxifen because of a possible adverse event (uterine cancer) that could potentially occur in the future.

These examples indicate that methods are required to eliminate such false positives. The Detec’t project developed by Kappa Santé [22] is an adverse drug reaction monitoring program based on data mining and statistical analysis techniques using social media texts. Our intent at this point was to distinguish between potential ADRs and non-ADRs among the disorders associated with a drug in messages from social media. In this paper, we investigate whether the distance between the terms representing drugs and disorders in the messages may help distinguish between ADRs and false positives.

### Related Work

The current technological challenges include the difficulty for text mining algorithms to interpret patient lay vocabulary [23].

After the review of multiple approaches, Sarker et al [9] concluded that following data collection, filtering was a real challenge. Filtering methods are likely to aid in the ADR detection process by removing most irrelevant information. Based on our review of prior research, two types of filtering methods can be used: semantic approaches and statistical approaches.

Semantic filtering relies on semantic information, for example, negation rules and vocabularies, to identify messages not corresponding to an ADR declaration. Liu and al [24] developed negation rules and incorporated linguistic and medical knowledge bases in their algorithms to filter out negated ADRs, then remove drug indications and non- and unreported cases on FAERS (FDA’s Adverse Event Reporting System) database. In their use case of 1822 discussions about beta blockers, 71% of the related medical events were adverse drug events, 20% were drug indications, and 9% were negated adverse drug events.

Powell et al [25] developed “Social Media Listening,” a tool to augment postmarketing safety. This tool consisted on the removal of questionable Internet pharmacy advertisements (named “Junk”), posts in which a drug was discussed (named “mention”), posts in which a potential event was discussed (called “Proto-AE”), and any type of medical interaction description (called “Health System Interaction”). Their study revealed that only 26% of the considered posts contained relevant information. The distribution of post classifications by social media source varied considerably among drugs. Between 11% (7/63) and 50.5% (100/198) of the posts contained Proto-AEs (between 3.2% (4/123) and 33.64% (726/2158) for over-the-counter products). The final step was a manual evaluation.

The second type of filtering was based on statistical approaches using the topic models method [26]. Yang et al [27] used latent Dirichlet allocation probabilistic modeling [28] to filter topics and thereby reduce the dataset to a cluster of posts to evoke an ADR declaration. This method was evaluated by the comparison of 4 benchmark methods (example adaption for text categorization [EAT], positive examples and negative examples labeling heuristics [PNLH], active semisupervised clustering based two-stage text classification [ACTC], and Laplacian SVM) and the calculation of *F* scores (the harmonic mean of precision and recall) on ADRs posts. These 4 methods were improved by the use of this approach. The *F* score gains fluctuated between 1.94% and 6.14%. Sarker and Gonzalez [16] improved their ADR detection method by using different features for filtering. These multiple features were selected by the use of leave-one-out classification scores and were evaluated with accuracy and *F* scores. These features were based on n-grams (accuracy 82.6%, *F* score 0.654), computing the Tf-idf values for the semantic types (accuracy 82.6%, *F* score 0.652), polarity of sentences (accuracy 84.0%, *F* score 0.669), the positive or negative outcome (accuracy 83.9%, *F* score 0.665), ADR lexicon match (accuracy 83.5%, *F* score 0.659), sentiment analysis in posts (accuracy 82.0%), and filtering by topics (accuracy 83.7%, *F* score 0.670) for filtering posts without mention of ADRs. The use of all features for the filtering process provided an accuracy of 83.6% and an *F* score of 0.678. Bian et al [29] utilized SVM to filter the noise in tweets. Their motivation for classifying tweets arose from the fact that most posts were not associated with ADRs; thus, filtering out nonrelevant posts was crucial.

Wei and al [30] performed an automatic chemical-diseases relation extraction on a corpus of PubMed articles. Their process was divided in two subtasks. The first one was a disease named entity recognition (DNER) subtask based on the 1500 PubMed titles and abstracts. The second subtask was a chemical-induced disease (CID) relation extraction (on the same corpus as DNER subtask). Chemicals and diseases were described utilizing the medical subject headings (MeSH) controlled vocabulary. They evaluated several approaches and obtained an average precision, recall, and standard *F* score of 78.99%, 74.81%, and 76.03%, respectively for DNER step and an average of 43.37% of *F* score with the CID step. The best result for CID step was obtained by combining two SVM approaches.

### Objective

We propose adding a filter based on Gaussian mixtures models to reduce the burden of other entities, that is, disorders that are mentioned in the messages but are not ADRs. The objective was to optimize ADR detection by reducing the number of false positives. We hypothesized that the shorter the distance between the disorder name and the drug, the higher the probability to be an ADR. The approach was applied to the Detec’t corpus.

## Methods

### Materials

#### Detec’t Database

We used a version of the Detec’t database that contained 17,703,834 messages corresponding to 350 drugs. The messages were extracted from 20 general health forums, all in French, using a custom Web crawler to browse the selected forums and scrape messages. The forums scraped do not restrict users with a limited number of characters in the message. Detec’t contains the messages extracted and associated metadata, namely users’ aliases and dates.

The Detec’t database was created in 2012 by Kappa Santé [22], a contract research organization founded in 2003 that specialized in post marketing studies and pharmacoepidemiology. Kappa Santé developed Detec’t to achieve this goal.

The messages that constitute our dataset came from (1) doctissimo, (2) atoute.org, (3) e-santé, (4) santé médecine, and (5) aufeminin. These are popular general forums dedicated to health with an average of 89,987 unique visitors a day in 2016. Users must register to be able to post a message in these forums.

#### Dataset Constitution

We randomly extracted 700 messages from the Detec’t database related to 3 drugs from 3 different therapeutic classes: Teriflunomide, Insulin Glargine, and Zolpidem.

**Figure 1:**
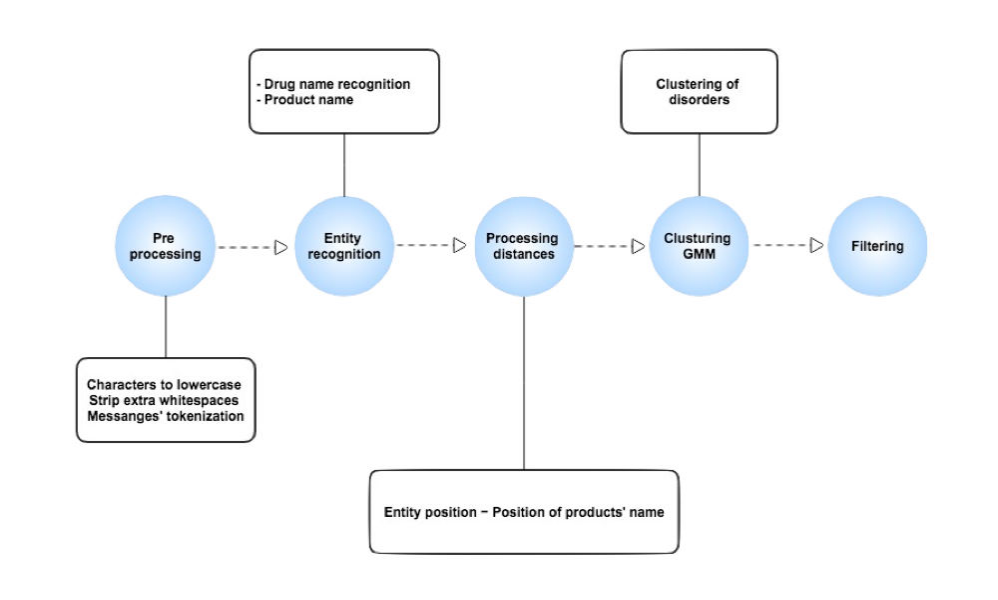
Summary diagram.

Of these, 52 messages did not contain any disease entity and were removed from the list. The remaining 648 messages were both manually annotated and automatically processed. Processing was performed in 5 steps; the method is summarized in Figure 1.

#### Medical Terminology

Regarding disorders, we used the Medical Dictionary for Regulatory Activities dictionary (MedDRA), which is the international medical terminology developed under the auspices of the International Conference on Harmonization of Technical Requirements for Registration of Pharmaceuticals for Human Use (ICH). The MedDRA dictionary is organized by system organ class (SOC) and divided into high-level group terms (HLGT), high-level terms (HLT), preferred terms (PT), and lowest-level terms (LLT). Synonymous LLT are grouped under a unique identifier labeled as preferred terms (PT).

We used a lexicon built in-house by Kappa Santé. The lexicon was derived from the French version of MedDRA 15.0 and was extended by adding more lay medical vocabulary. A fuzzy grouping technique was used to group commonly misspelled words or closely spelled words under one term. The grouping was performed at the MedDRA LLT level. The fuzzy grouping algorithm temporarily strips all vowels (except the first one), strips double or triple consonants from extracted words, and then compares them to see if they are the same. For example, “modeling” and “modelling” would be grouped together [31-34]. The original 15.0 release of MedDRA contains 19,550 PT and 70,177 LLT. Our lexicon contained a total of 19,530 PT and 63,392 LLT. Among them, 259 additional LLT were added by Kappa Santé, for example, “mal au crâne” (French familiar broadly used expression for headache) as a synonym of “mal de tête” (headache). Although not pure synonyms, as “crâne” is not equivalent to “head,” “mal de crâne is a familiar broadly used expression for headache. The decrease in the number of terms was caused by the removal of some PTs that were beyond the scope of ADRs, such as “married.” Moreover, the lexicon was manually reduced by grouping terms with similar meanings, for example, the PTs for “alcoholic” and “alcoholism.” Our final version for disorders contained a total of 63,392 terms (LLT level), including both original MedDRA LLT terms and nonstandard terms. We used the most specific (LLT) level to search for disorder entities in the posts.

#### Manual Annotation

An ADR is a sign or symptom caused by taking a medication. ADRs may occur following a single dose or prolonged administration of a drug or result from the combination of 2 or more drugs.

A disorder concept corresponds to a sign or symptom, a disease, or any pathological finding. In the context of a message, a disorder may:

- Either play the role of an adverse event, (ADR) for example, “I took aspirin, it gave me a terrible headache.” These are considered “true ADRs.”
- Or correspond to a condition that is not reported by the patient as an ADR, for example, “I had a headache so I took aspirin.”

With the objective of distinguish between ADRs and disorders that were not ADRs, 2 experts manually annotated the messages to identify true ADRs.

The annotators labeled each disorder entity in the messages as (1) « ADR » if the patient reported the disorder as a possible ADR in his or her message, or (2) « other entity » if the disorder was not reported as an ADR in the message.

This annotated dataset was used as a gold standard.

### Analysis Phases

#### Data Preprocessing

**Figure 2:**
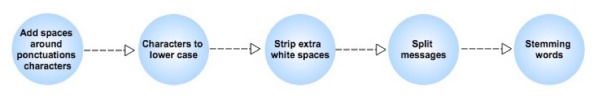
Data preprocessing steps.

The standardization of our approach required preprocessing the dataset to avoid some cases of poor data quality. Figure 2 presents these preprocessing steps.

The character separation method involved inserting whitespaces around every punctuation character. This separation was necessary due to the poor data quality to optimize disorder identification.

Because we used the R software (a language and environment for statistical computing provide by the R core team in Vienna) to process and analyze data, and given that R discriminates between lowercase and uppercase words, we used the “tm” Package (a text mining framework for R software) to convert the document text to lower case and remove extra whitespaces [35]. We did not remove stop words because our hypothesis was based on the number of words separating drug names and disorder terms. The stop words removing could have impacted the distances distribution.

The last step was the tokenization of messages. Word segmentation provides a list of words in each message and their positions in the post.

#### Named Entity Recognition

The objective of the named entity recognition module was to identify 2 types of entities in a patient’s post: drug names and disorders.

As the extended lexicon for disorders that we developed contained colloquial terms as well as expressions with spelling and/or grammatical errors, lexicon matching was performed using exact match methods after stemming of both messages and expressions in the lexicon.

Drug names in the messages were automatically identified using fuzzy matching and stored in the Detec’t database as message metadata. To minimize the impact of misspelled words, each word was first stemmed using a Porter stemmer, an algorithm meant to remove inflection from a word [12,16]. Savoy [36] demonstrates that the use of Porter stemmer algorithm improved information retrieval by 30.5% with French language.

All of the other terms in the messages were mapped to our extended version of MedDRA, which includes colloquialisms, abbreviations, and words with spelling errors. Lexicon matching was implemented as string matching using regular expressions. The granularity of the disorder concepts extracted from the messages corresponded to the LLT level of MedDRA.

#### Processing Distance Between Entities

After the preprocessing step, the position of each entity in the message was calculated. We defined a word as a continuous series of characters between 2 whitespaces. The distance between a drug “a” and a disorder “b” in a message was defined as the number of words separating the two entities:

Distance (a,b)=(position of b) − (position of a)

The following data were automatically collected:

- The disorder name (corresponding to b) detected in the message and the corresponding LLT.
- The MedDRA preferred term associated to the disorder term.
- The overall position of the disorder term in the message.
- Relative position of the detected disorder to the product’s name (before or after).
- The distance between the disorder term and the drug name.
- Length of the message (expressed in number of words)

When the product name appears several times in a message, the algorithm evaluates the distances between a disorder and all drug name occurrences. The pairs identified are deduplicated. The only pair considered is the one that minimizes the distance to the drug name.

#### Clustering Method

**Figure 3:**
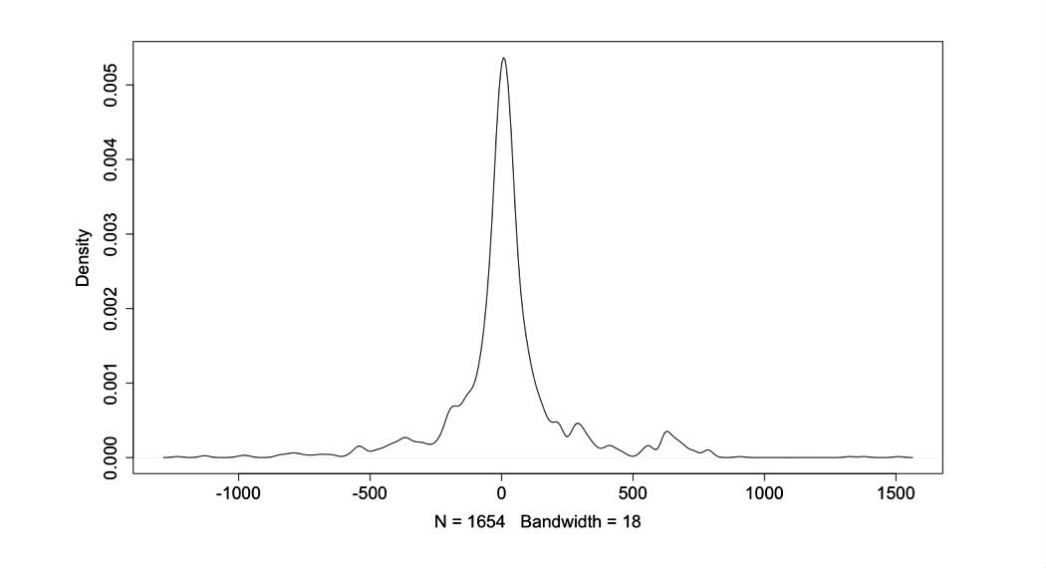
Observed density of distances between disorder terms and drug names.

We used Gaussian mixture models for the disorder clustering using “mclust” R package [37]. We modeled ADRs and other entities as normal distributions mixed on one. The global distribution is obtained by modeling distances calculated for each disorder (Figure 3). EM algorithm is used for model fitting. The affiliation of each type of entity is established by the use of likelihood maximization.

## Results

### Descriptive Analysis

**Figure 4:**
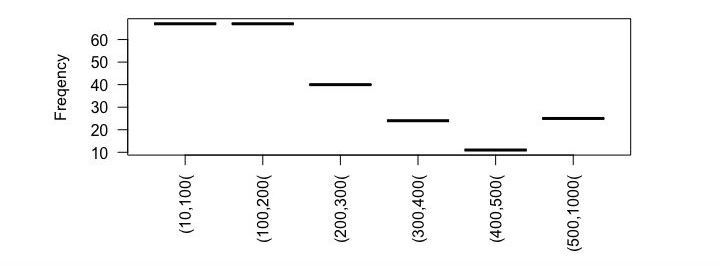
Documents under review found consisting of (n) words.

We processed a total of 648 messages from 5 French forums written from 2002 to 2013. The named entity recognition module automatically identified 320 unique disorders corresponding to 268 PTs (see Multimedia Appendix 1 for an exhaustive list of disorders identified). Among the 648 messages, 40.9% (265/648) contained drug names but no disorder term. The automatic processing was able to extract 1654 (drug and disorder) pairs from the 383 messages containing at least one disorder term. Figure 4 shows the number of messages consisting of (n1, n2) words. Nine messages contain more than 1000 words.

**Figure 5:**
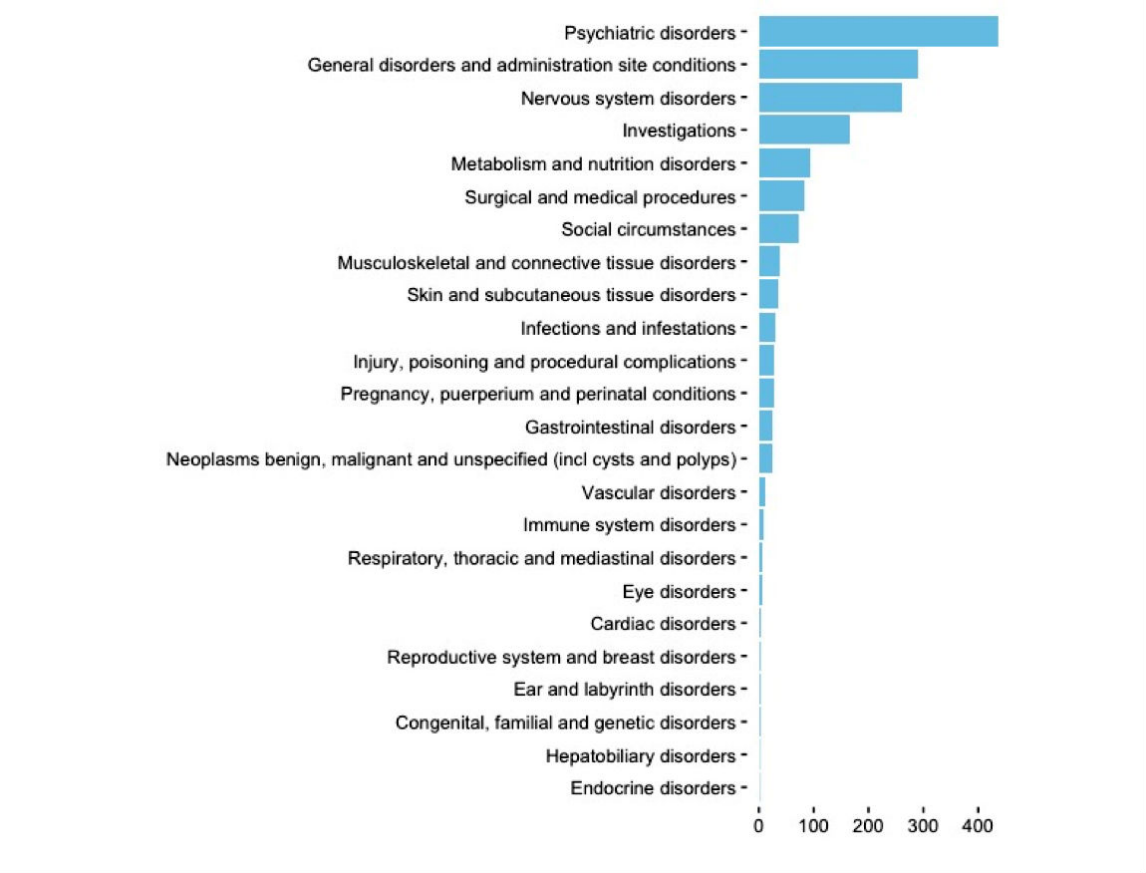
Disorders automatically identifies (MedDRA system organ class [SOC] level).

All 1654 of the identified disorders were manually annotated as true ADRs or not. Among them, the experts identified 11.42% (189/1654) of ADRs and 88.57% (1465/1654) of other entities. Figure 5 shows the disorders found in the messages grouped at SOC level of MedDRA.

### Analysis of Disorder Entity Distribution

As shown in Figure 3, the distribution of the distances between disorder terms and drug names in the messages seems to follow a Gaussian distribution. However, a Shapiro-Wilk normality test significantly rejected the null-hypothesis with a *P* value of less than 2.2e-16.

**Figure 6:**
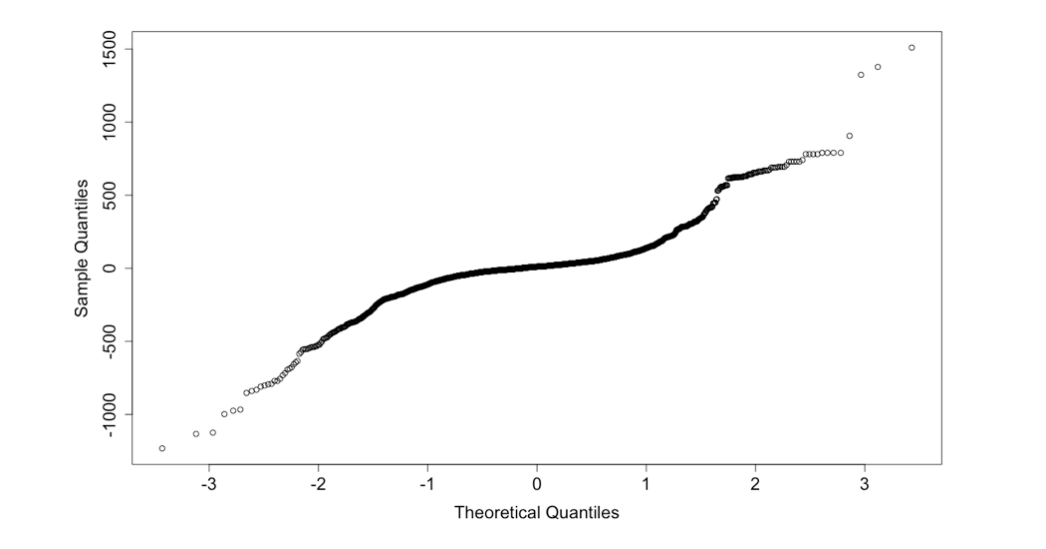
Normal Q-Q plot.

QQ-plot in Figure 6 shows that the data are heterogeneous and can be a mixture of multiple Gaussian distributions [38,39].

The clustering method clusters the detected disorder concepts based on their distances (expressed as a number of words) to the product name in each message. To achieve this goal, we used Gaussian mixture models and EM algorithm [40-42].

**Figure 7:**
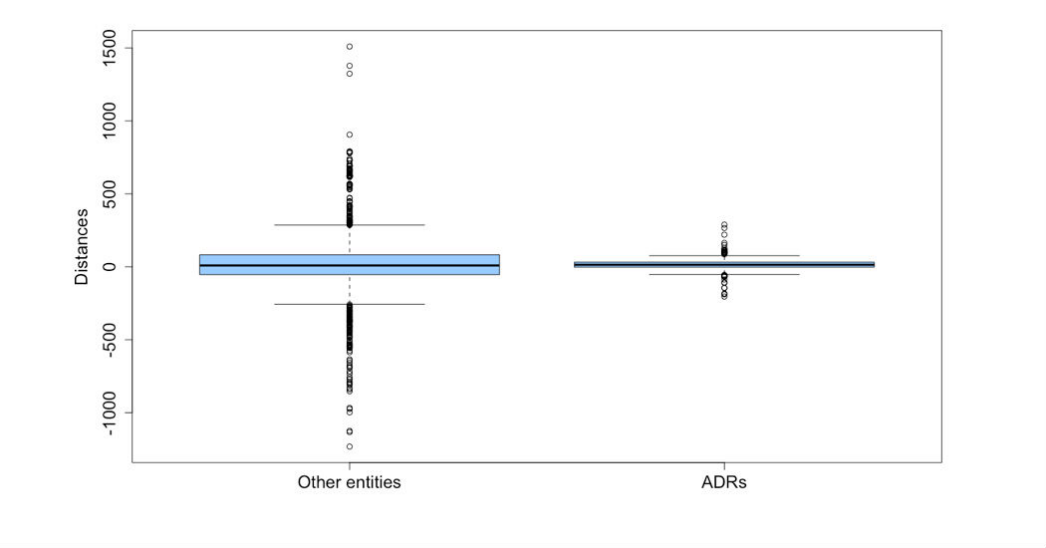
Repartition of true adverse drug reactions (ADRs) and other entities by distances.

Distance distribution also varies greatly with a viewing averaging 20.32 and a median value of 11.0. The distances vary between 1233 before the drug name and 1510 after. Figure 7 shows the concentration of ADRs in a short interval around drug names. The ADRs are contained in an interval between 204 words before product names and 289 after words around drug names. All disorders located beyond 289 words are not ADRs (false positives).

### Clusters Analysis

**Figure 8:**
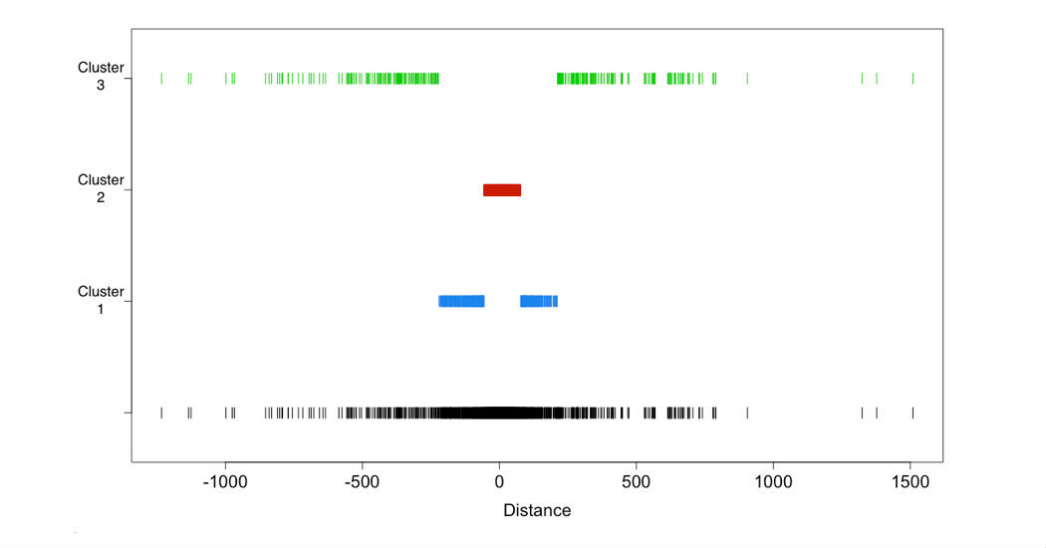
Supervised clustering results.

We applied a supervised clustering method with three fixed clusters (Figure 8).

Cluster 1 corresponds to distances in the (−220, −57) union (+78, +211) interval, that is, between 220 and 57 words before the drug name or in the interval between 78 and 211 words after the drug name. Cluster 1 contains 441 disorders. Among them, 6.6% (29/441) of the disorders found in cluster, are true ADRs.

Cluster 2 corresponds to distances in the (−56, −1) union (+2, +77) interval (ie, between 56 and 1 words before the drug name or between 2 and 77 words after the drug name). Cluster 2 contains 889 disorders. In cluster 2, 17.7% (157/889) of the disorders are true ADRs.

**Table 1:** Supervised clustering contingency table.

Clusters	Other entities (%)	ADRs^a^ (%)	Total
Cluster 1	412 (93.4)	29 (6.6)	441
Cluster 2	732 (82.3)	157 (17.7)	889
Cluster 3	321 (99.1)	3 (0.9)	324
Total	1465	189	1654

^a^ADRs: adverse drug reactions.

Cluster 3 corresponds to distances between 1233 and 222 words before the drug name or between 212 and 1510 words after. Cluster 3 contains 324 disorders. Among them, 0.9% (3/324) are ADRs and 321 are other entities.

### Filtering Strategies

We tested two filtering strategies. The objective was to filter out the entities that were not ADRs. Table 1 shows supervised clustering contingency.

In the first filtering strategy, we merged clusters 1 and 3 (Table 2). The disorders in clusters 1 and 3 (Table 1) are in the (−1233, −57) union (+78, +1510) interval. The objective of this strategy was to maximize the number of disorders that are not ADRs (412 in cluster 1 and 321 in cluster 3) in one cluster for filtering. The union of these 2 clusters contained only 4.2% (32/765) of ADRs. As shown in Table 2, 95.8% (733/765) of the disorders that are present in the union of clusters 1 and 3 correspond to disorders that are not ADRs (733 disorders).

**Table 2:** Filtering by merging of clusters 1 and 3.

Clusters	Other entities (%)	ADRs^a^ (%)	Total
Clusters 1 and 3	733 (95.8)	32 (4.2)	765
Cluster 2	732 (82.3)	157 (17.7)	889
Total	1465	189	1654

^a^ADRs: adverse drug reactions.

In the context of ADR detection, the use of this approach to remove disorders of clusters 1 and 3 induces a 50.03% reduction of potential false positives.

The ability to detect false ADRs achieved a precision score of 95.8% and a recall of 50.0%. In other terms, almost all (>95%) of the pairs that were filtered out were not true ADRs, but the system detected only 50.03% (733/1465) of the false positives.

A second filtering strategy involved merging clusters 1 and 2 (Table 3). The main objective of this strategy was to aggregate as many ADRs as possible. We used the union of clusters 1 and 2 (412 disorders in cluster 1 and 732 disorders in cluster 2) and then filtered out the disorders from cluster 3.

**Table 3:** Filtering by merging clusters 1 and 2.

Clusters	Other entities (%)	ADRs^a^ (%)	Total
Cluster 3	321 (99.1)	3 (0.9)	324
Clusters 1 and 2	1144 (86.0)	186 (14.0)	1330
Total	1465	189	1654

^a^ADRs: adverse drug reactions.

The union of clusters 1 and 2 contains 98.4% (186/189) of the true ADRs present in the dataset. Given that cluster 3 contains only 1.6% (3/189) of the ADRs in our study, exclusion of cluster 3 leads to erroneously ignoring only three relevant adverse events.

Using this filtering strategy, our detection of disorders that are not ADRs achieved a precision of 99.07% and a recall of 21.9%. In other terms, 99.07% (321/324) of the pairs that were filtered out were false positive, but the system detected only 21.91% (321/1465) of the non-ADRs.

## Discussion

### Principal Findings

We demonstrated that the meaning of a disorder term in a message varies considerably based on its distance to the drug name. Noticeably, before any filtering strategy, cluster 3 contained only three ADRs. The higher the distance between the disorder and the drug name is, the lower the probability that the disorder might be an ADR. Specifically, in cluster 3, 99.1% (321/324) of the disorder terms did not correspond to ADRs. Our approach based on distance measurement enabled us to filter out other (non-ADRs) entities from the detected disorders. The first strategy enabled us to automatically filter out 49.96% (732/1465) of the disorders that were not ADRs. The second strategy filtered out 78.08% (1144/1465) of the disorders that were not ADRs. Consequently, we obtained a significant improvement in identifying non-ADRs (false positives) in messages. Such filtering can be used as a first step to optimize the screening of ADRs by reducing the false positive rate.

### Comparison With Prior Work

Patient’s adverse drug event discussions in forums are more informal and colloquial than biomedical literature and clinical notes. When messages in social media are mined to detect ADRs declarations, these informal chats lead to many noisy false positives. The use of filtering methods improves ADR detection in the huge data source that is social media [16,25,27]. Powell et al [25] showed that only 26% of such posts contain relevant information. Even when a message contains both a drug name and a disorder term, the latter may play a role other than an ADR. In our dataset, only 11.42% (189/1654) of the disorder terms corresponded to potential ADRs.

However, the use of distance (as number of words) has not been used for ADR detection, and the usage of this type of information for ADR classification is novel. Sarker and Gonzalez [16] used a leave-one-out classification to evaluate different features for a filtering approach. One of these approaches is based on the n-gram method (accuracy 80.7%), and another approach is based on topic evaluation (accuracy 86.1%). Our approach is different and can be combined with other filtering methods.

One challenge is the comparison of the different filtering methods and their evaluation on equivalent datasets. We evaluated our method on a corpus that was not specific to an adverse event. We relied on MedDRA, which encompasses the complete spectrum of possible ADRs.

### Limitations and Future Work

Some limitations regarding the effectiveness of our filtering method should be noted. The main limitation is that our classification process is less efficient when the disorder term is closer to the drug name in the message. Another limitation is that the distance approach has been developed and tested on a French corpus and must be adapted to different languages. Finally, this approach is based on the number of words between drug names and disorder entities in messages and is therefore not applicable to some forms of social media such as Twitter because a tweet would not contain a sufficient number of words to satisfy a sufficient disparity of the disorders detected. The insufficient disparity would not allow our filter to effectively classify the disorders.

Many patients express sentiments when posting about drug associated events in social media, and (quoting Sarker and Gonzalez in [16]) “the sentiments generally correlate strongly with the reactions associated with the drugs they are taking.” Combining lexical features from research areas such as sentiment analysis or polarity classification with methods that detect ADRs can improve the automatic classification of ADR mentions from social media text. Moreover, it can help analyzing consumer’s perceptions and their changes in time, for example, following media coverage.

### Conclusions

We have demonstrated that the distance between the disorder and the drug in a message influences the probability of a disorder to be a genuine ADR. The use of distance between entities on patient posts from social media enabled us to filter out false positives from the detected disorders, and thus, to optimize ADR screening.

## Appendix

Multimedia Appendix 1 Exhaustive list of disorders found at MedDRA preferred terms (PT) and system organ class (SOC) levels.

## Abbreviations

ACTC: active semisupervised clustering based two-stage text classification
ADR: adverse drug reaction
AE: adverse event
EAT: example adaption for text categorization
EM: expectation-maximization
FAERS: FDA’s Adverse Event Reporting System
HLGT: high-level group terms
HLT: high-level terms
LLT: lowest-level terms
ME: Maximum Entropy
MedDRA: Medical Dictionary for Regulatory Activities
MeSH: medical subject headings
NB: Naïve Bayes
PNLH: positive examples and negative examples labeling heuristics
PT: preferred terms
SOC: system organ class
SVM: support vector machine
